# Nest Initiation in Three North American Bumble Bees (*Bombus*): Gyne Number and Presence of Honey Bee Workers Influence Establishment Success and Colony Size

**DOI:** 10.1673/031.010.13001

**Published:** 2010-08-11

**Authors:** James P. Strange

**Affiliations:** Pollinating Insect Research Unit, USDA-ARS, 255 BNR, Utah State University, Logan, Utah, USA, 84322-5310

**Keywords:** bumble bees, nesting success, colony parameters, nest initiation

## Abstract

Three species of bumble bees, *Bombus appositus* Cresson, *Bombus bifarius*, Cresson and *Bombus centralis* Cresson (Hymenoptera: Apidae) were evaluated for nest initiation success under three sets of initial conditions. In the spring, gynes of each species were caught in the wild and introduced to nest boxes in one of three ways. Gynes were either introduced in conspecific pairs, singly with two honey bees, *Apis mellifera* L. (Hymenoptera: Apidae) workers, or alone. Nesting success and colony growth parameters were measured to understand the effects of the various treatments on nest establishment. Colonies initiated from pairs of conspecific gynes were most successful in producing worker bees (59.1%), less successful were colonies initiated with honey bee workers (33.3%), and least successful were bumble bee gynes initiating colonies alone (16.7%). There was a negative correlation between the numbers of days to the emergence of the first worker in a colony to the attainment of ultimate colony size, indicating that gynes that have not commenced oviposition in 21 days are unlikely to result in colonies exceeding 50 workers. *B. appositus* had the highest rate of nest establishment followed by *B. bifarius* and *B. centralis.* Nest establishment rates in three western bumble bee species can be increased dramatically by the addition of either honey bee workers or a second gyne to nesting boxes at colony initiation.

## Introduction

Bumble bees (Hymenoptera: Apidae) are important pollinators of crops and wild land plants and have become the primary pollinators for crops in protected cultivation (Velthuis and van Doom 2006). Until the late 1990s, the primary species of bumble bee managed for pollination in western North America was *Bombus occidentalis* Greene (Whittington and Winston 2004; Velthuis and van Doom 2006), a native of western North America. However, due to disease problems in the species, production shifted to *B. impatiens* Cresson (Whittington and Winston 2004; Velthuis and van Doom 2006), a native to eastern North America. Since the early 2000s, *B. impatiens* has been the only bumble bee widely available for purchase in the United States and Canada. Despite the ubiquity of *B. impatiens* in commercial operations, many other bumble bees can be successfully reared in captivity and some species show commercial promise [e.g., *B. vosnesenskii* Radoszkowski (Dogterom et al. 1998), and *B. occidentalis* (Whittington and Winston 2004)] if mass production issues can be rectified.

Because *B. impatiens* is an eastern North American species, concern has been expressed about the prudence of using the species west of the Rocky Mountains (Whittington and Winston 2003, 2004; Colla et al. 2006; Velthuis and van Doom 2006). This concern has been underscored by recent work suggesting that commercially produced colonies placed in greenhouses can lead to pathogen dispersal into wild populations in the vicinity of the greenhouse operations (Colla et al. 2006; Otterstatter and Thompson 2008). The collapse of commercial *B. occidentalis* populations and the possible extinction of *B. franklini* in Oregon and California (Thorp et al. 2003) have added to concerns about pathogen spread through bumble bee transport (Evans et al. 2008). In response to these concerns, several states have placed restrictions on importing non-native bumble bees for pollination, including Oregon's ban on importation of non-native species for use in greenhouses or open field pollination (Anonymous 2009) and California's prohibition of open field releases of *B. impatiens* (Anonymous 2008). Because of restrictions like these and growing interest in protected cultivation, having a western native bumble bee for crop pollination is becoming increasingly important.

Several considerations must be made when developing a pollinator for commercialization, including disease and pest problems, effective pollination of the target crop, and ease of management (Macfarlane et al. 1994). A major obstacle to developing bumble bee species as a commercially viable pollinator is accommodating the the annual life cycle of the colony (Sladen 1912; reviewed by Velthuis and van Doom 2006). While bumble bees are social and some species can form nests with over a thousand individuals (Plowright and Jay 1966; Johansen 1967; Macfarlane et al. 1994), temperate species do not form perennial colonies. Instead, the annual colony cycle begins when a mated gyne (-queen) emerges from winter dormancy and searches for a suitable nesting site. She then forages to provision her nest, oviposits, and incubates her first brood clutch. After emergence of the first brood, she then restricts her activity to oviposition and brooding, whereas her offspring perform the worker tasks of foraging, brood care, and colony maintenance. Thus, establishing a year round production of bumble bees is necessarily dependent on first establishing a large number of nests from wild-caught gynes that can serve as sources for future reproductive males and gynes to be used in commercial colony production (Röseler 1985; Velthuis and van Doom 2006).

Despite previous work on nest establishment, success rates can be low when working with lab-reared or wild-caught gynes. Of 24 labreared *B. terrestris* gynes introduced into boxes without a nesting stimulator, Kwon et al. (2006) observed only 6 that produced a first brood worker. Mah et al. (2001) reported between 25% and 48.9 % successful nest establishments with wild-caught gynes of three Korean *Bombus* species. Given the biological variability among species, methodology must be tested on each individual taxon (Plowright and Jay 1966; Mah et al. 2001; Kwon et al. 2006; Yoneda 2008) in order to maximize species-specific nesting success. To establish laboratory populations for pollination studies and experimental manipulations, maximizing nesting success of wild-caught gynes is important. While it is not always clear what factors are most critical for explaining nesting failure, it can be related to poor mating or presence of disease in the young gynes (Velthuis and van Doom 2006). Another possibility is that establishing a nest is energetically difficult as the new gyne must build and provision a honey pot and build brood cells for her offspring (Heinrich 2004), and a single foundress has difficulty meeting the energy requirements of nest establishment.

To increase nesting success, various methods have been tried using a variety of *Bombus* species as test subjects. An increase in nesting success has been demonstrated when two gynes were used to establish colonies (Sladen 1912; Ptáček et al. 2000). This phenomenon of cofounding (or pleometrosis) is known from *Polistes* wasps and may be adaptive in forming new colonies in primitively eusocial Hymenoptera (Hunt 2007) and ants (reviewed in Hölldobler and Wilson 1977). In *Bombus*, pleometrosis has been shown to increase oviposition success in some eastern North American bumble bee species (Plowright and Jay 1966); however, it was not reported if cofounding increased the success of rearing adult offspring or simply the incidences of oviposition. The presence of worker honey bees, *Apis mellifera* L. (Hymenoptera: Apidae) during nest establishment has been shown to increase nesting success in *B. terrestris* L. and *B. pascuorum* (Scopoli) colonies (Ptáček and Drobna 2006; Velthuis and van Doom 2006). The use of conspecific workers of *B. terrestris* in the presence of frozen conspecific pupae (Kwon et al. 2006), the presence of fresh cocoons (Kwon et al. 2003; Yoneda 2008), and older cocoon material (Velthuis and van Doom 2006) have all been demonstrated as methods to increase success in initiating colonies from lab-reared gynes.

Each system of nest establishment has limitations especially when attempting to rear colonies from wild-caught gynes whose availability may be sporadic and limited. One limitation to employing workers is the unavailability of young worker honey bees or bumble bees in the temperate region during the winter or early spring, especially for a small scale bee producer. As noted by Kwon et al. (2006), additions of frozen pupae, cocoons, or previously used nesting material necessitate storage of these items and presume previous success in rearing bumble bee colonies. Yoneda (2008) reared colonies of *B. terrestris* alongside colonies of other species to provide fresh cocoon material for rearing experiments. Because of the relatively low rate of nesting success in the laboratory that is typically observed when starting nests from wild-caught gynes, the present study tests two alternatives to the single gyne method described in Plowright and Jay (1966) and Evans et al. (2007) with minor modifications described below.

Three species of bumble bees that are widely distributed in the western United States are *Bombus appositus* Cresson, *Bombus bifarius* Cresson and *Bombus centralis* Cresson. *B. appositus* is in the subgenus *Subterraneobombus*, which is in the long faced bee clade (Cameron et al. 2007). It is a large-bodied species distributed throughout the forested mountains of the western US and Canada. In northern Utah it is common at elevations over 1800 m and encountered more rarely at lower elevations, forming nests with typically fewer than 100 individuals (Hobbs 1966). *B. bifarius* and *B. centralis* are members of the subgenus *Pyrobombus*, in the short faced clade (Cameron et al. 2007). Both species are smaller-bodied than *B. appositus*, and each species can produce colonies of several hundred individuals (Hobbs 1967; JP Strange, unpublished observations). Both *B. bifarius* and *B. centralis* are commonly found in Utah between 1400 m and 3000 m; although, *B. centralis* generally occurs at the lower end of that zone and *B. bifarius* at the upper elevations (unpublished oservations).

To test the hypotheses that cofounding and single gyne founding in the presence of honey bee workers increase nest establishment in these three bumble bee species, the effects of nest starting conditions on nest development was examined using wild-caught gynes of *B. appositus, B. bifarius*, and *B. centralis.* Nest establishment was defined as the production of at least one live adult worker by the gyne(s).

## Materials and Methods

**Figure 1.**
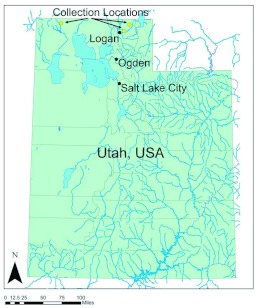
Map of the state of Utah (USA) with spring gyne collection locations represented by yellow dots and cities represented by black dots. High quality figures are available online.

In the spring of 2008, bumble bee gynes emerging from winter dormancy were netcollected while nest searching or nectar foraging on flowers. Gynes with pollen in their corbiculae were immediately released as that is a sign that they have already established nests in the wild (Evans et al. 2007). Gynes were captured at several locations in Cache County and Box Elder County in northern Utah (Fig. 1). A total of 15 *B. appositus*, 54 *B. bifarius*, and 29 *B. centralis* gynes were captured. Upon removal from the net, bees were transferred to 10 dram plastic collection vials and stored in the dark for up to 2 h, whereupon they were transferred into 15 × 15 × 10 cm wooden holding boxes (one box per species) provisioned with 6 ml 50% sugar syrup feeders for transport to the laboratory; holding boxes held up to 35 gynes. Newly captured *B. appositus* gynes were placed in boxes of no more than 10 individuals to reduce fighting, whereas gynes of the other species were not observed fighting and thus this treatment was not required. Upon arrival at the laboratory, gynes were held in the boxes for 24 h in the dark at 26–30° C and relative humidity 40–60% until they were transferred into individual nesting boxes.

Nesting boxes were 10 × 15 × 10 cm corrugated plastic boxes with 2.5 cm ventilation holes at the longitudinal ends covered with 8-mesh hardware cloth. The floor of each box was covered with a layer of 0.6 cm plastic mesh and the lid was a 10 × 15 cm piece of clear plastic, taped (in a hinge fashion) to the box. Each box was provisioned with a feeder containing 6 ml of 50% inverted sugar syrup in water, a wax cup (approximately 1 ml) fabricated from beeswax, also provisioned with inverted sugar syrup, and a 1.0 g pellet of pollen coated with beeswax to arrest dehydration (Evans et al. 2007). Inverted sugar was used to prevent crystallization in feeders.

To transfer gynes from the holding boxes to the nesting boxes, the gynes were first anesthetized with CO_2_ by inundating the sealed holding box with gas. Inundation was typically 30 sec, but was adjusted upwards in cases when the bees were not fully anesthetized (especially for the larger bodied *B. appositus*). When gynes were subdued, they were removed from the holding box with forceps and gently placed in the provisioned nesting boxes. This technique was preferred to chilling due to the evidence that CO_2_ stimulates oviposition in *B. terrestris* (Röseler 1985).

Two treatments and a control were used to study nest initiation, and usually about onethird of the collected gynes was used per treatment. The control was the placement of a single gyne into a nesting box with no honey bee workers. The second treatment involved placing a single *Bombus* gyne into a nesting box that contained two newly emerged *A. mellifera* workers. The third, cofounded, treatment involved placing two conspecific gynes into the nesting box together. After placement into the nesting boxes, the boxes were maintained in the dark and held at 26– 30° C and relative humidity 40–60% for three days without disturbance. After three days, pollen and sugar were provided as needed. Within the first three days following introduction to the nest box, dead honey bee workers were replaced with new workers, but after day 3, honey bees that died were not replaced.

Nest boxes were assessed under red light to avoid disturbing colonies. Each nest box was observed daily for the duration of the experiment. Nesting success was defined as the ability of a gyne to produce at least one adult female (worker) offspring. Days to first offspring, days to 20 workers, maximum colony population, and colony longevity were recorded.

### Statistical analysis

Data were transformed using the logY + 1 transformation to conform to the assumptions of the analysis. General Linear Model Analyses of Variance were run to compare treatment results using the test parameter as the dependent variable, and treatment and species as the fixed factors. Nesting success was scored as 0 (no offspring) or 1 (≥1 worker produced) and analyzed using the nonparametric Mann-Whitney U test on pairs of treatments and pairs of species. To study the effect of nest establishment on the rate of colony development and mature colony size, the days to first worker production were correlated to the days to 20 workers and maximum colony size within each nest using Pearson's correlation. Significance for all comparisons was set at the P < 0.05 level. Statistical computations were performed using SPSS v. 15 (SPSS 2006).

## Results

Of the three species studied, *B. appositus* gynes produced at least one worker (the minimum criterion of successful nesting) in 53.8% of the attempts to establish colonies from wild caught gynes (Table 1). Singlegyne nests without honey bee workers successfully nested 2/6 times (33.3%) (Table 2), gynes with honey bee worker helpers nested 3/5 times (60%), and cofounded nests successfully nested 2/2 times (100%).

**Table 1.**
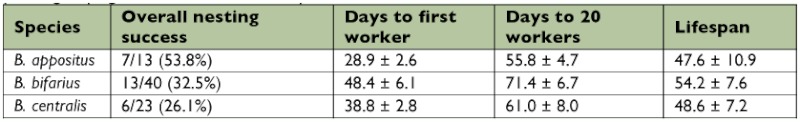
Nesting success rates as defined by the production of one or more workers, days to the emergence of the first worker ± SEM, days to the emergence of the twentieth worker ± SEM and average colony lifespan in captivity for three *Bombus* species grouping results from three colony initiation treatments.

**Table 2.**
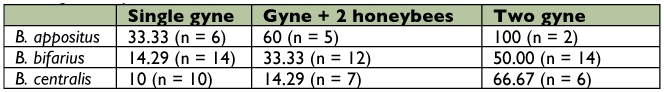
The percent of *Bombus* nests that produced at least one worker for each species by treatment and the control. Initial numbers of nests are given in parentheses.

Of 40 nests, *B. bifarius* successfully nested 32.5% of the time (Table 1). Single gynes with no honey bee workers successfully nested 2/14 (14.3%) times, single gynes with two honey bee workers nested 4/12 times (25%), and cofounded nests successfully produced workers in 7/14 attempts (50%) (Table 2).

Of 23 nests *B. centralis* gynes successfully nested 26.1% of the time (Table 1). Single gynes with no honey bee workers successfully nested 1/10 times (10%), single gynes with two honey bee workers nested 1/7 times (14.3%), and cofounded colonies successfully produced workers in 4/6 attempts (60%) (Table 2).

There was no significant difference in the nesting success among the three species (*F* = 2.339; df = 2, 75; *p* = 0.104) (Table 1); however, there was a significant effect of treatment on nesting success (*F* = 5.979; df = 2, 75; p = 0.004) (Table 3). Cofounded colonies produced offspring more frequently than colonies initiated with single gynes; however, there were no differences in successful nesting among any other treatment comparisons. Across the three species, colonies initiated with single gynes and no honey bees established nests 5 of 30 times (16.7%), colonies initiated with single gynes and honey bee workers produced offspring successfully 8 of 24 (33.3%) times, and colonies started with two gynes were successful 13 of 22 (59.1%) times (Table 3).

Despite the increased success of producing at least one offspring, nest establishment methodology had no significant effect on other colony growth parameters except the lifespan of the gynes (Table 3). In cofounded nests, at least one gyne lived significantly longer than nests initiated with either single gynes or single gynes with honey bee workers (*F* = 4.187; df = 2, 74; *p* = 0.019). No significant effect of treatment on the number of days to first worker production in successfully nested colonies (*F* = 0.090; df = 2, 25; *p* = 0.914) was found, nor was there a significant effect of species on the days to emergence of the first worker (*F* = 2.965; df = 2, 25; *p* = 0.079). Likewise, there was no effect of either species or treatment on the number of days until the nest population reached 20 workers (*F* = 1.245; df = 6, 13; *p* = 0.386).

**Table 3.**
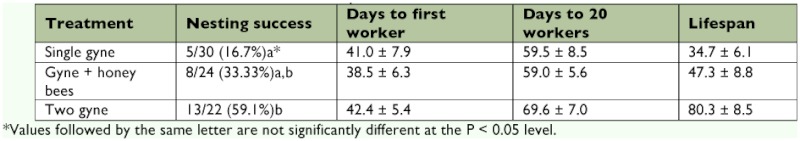
Nesting success rates as defined by the production of one or more workers, days to the emergence of the first worker ± SEM, days to the emergence of the twentieth worker ± SEM, and average colony lifespan in captivity for two colony initiation treatments and a control for three *Bombus* species.

**Table 4.**
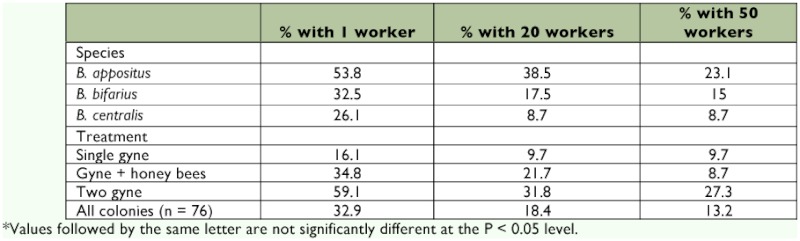
Percent of colonies of each species and within each treatment and the control that produced colonies of one worker. 20 workers and 50 workers.

While no significant effect of treatment on the time to first worker emergence was observed, a significant correlation between the number of days to first worker production and days to emergence of the twentieth worker was found (*R* = 0.590; *p* = 14; *p* = 0.026). Additionally, there was an inverse correlation of days to first worker production on maximum colony size (*R* = -0.539; n = 26; *p* = 0.005) indicating that a shorter time interval to first worker emergence resulted in a larger colony. In fact, only two colonies that exceeded 40 days to the first worker emergence resulted in colonies of over 15 individuals. Those two colonies had the first workers emerge on days 46 and 47, respectively. Of all the colonies of the three species that produced workers, only 13.2% produced colonies with more than 50 workers (Table 4).

## Discussion

Initiating nests from wild caught gynes of the three species studied is most effective when two gynes are placed together in the starter box. This situation resulted in a 1.7-fold increase in nesting rate over single gynes with workers and a 3.7-fold increase over unaided wild-caught gynes. However, because two gynes are used to establish nests the actual per gyne success rate of the two gyne system is similar to the honey bee aided system. While the present study did not test the possibility, it has further been suggested that the nesting pair of gynes can then be split and will form two nests in some species (Michael Juhl, commercial producer of B. vosnesenskii in Olympia WA, personal communication).

The exact mechanism underlying the increase in nesting success when starting with two gynes is unclear. It may be that the gynes are competing for nesting space and the first to rear brood commands the nest. Under that hypothesis, one would expect that nest initiation would be faster as the two gynes compete to rear offspring; however, the lack of a significant difference in days to the first brood between monogynic and polygynic nests suggests otherwise. Alternatively, the relationship of the two gynes may be more cooperative in nature until offspring are reared (in most cases one of the gynes died within a week before or after first worker emergence). This scenario seems to align with the *Polistes* model of multiple foundress colonies where cofoundresses are cooperative, but then form a dominance hierarchy (reviewed in Hunt 2007). The two *Bombus* gynes may be working together to build the honey pot, thermoregulate the nest or feed developing brood. However, as has been previously documented (Plowright and Jay 1966; reviewed in Sakagami 1976), the polygynic state was not maintained after offspring emerged indicating that any cooperative state is short-lived. The present study did not document behavioral changes at that stage, but generally, within a week of first worker emergence, one of the gynes was dead (presumably killed by the successful gyne). For that reason the terminology of gynes instead of queen, which implies that only one is producing workers, seems more apt when discussing *Bombus* nest establishment (Hölldobler and Wilson 1977).

Despite the increase in nesting success resulting from using two gynes, it may be more valuable to install individual gynes with honey bee workers for nest initiation. Because it requires twice as many gynes to establish two-gyne nests, it is only beneficial to use a cofounded system if it is more than twice as likely to result in nest establishment. Although there is no significant difference between the honey bee treatment and two gyne treatment in nesting success, the average nesting success with honey bees is intermediate between that of single and multiple gynes. In fact, the polygynic system was slightly less than twice as likely to establish a nest compared to single gynes with honey bee workers. From an initial number of 100 wild-caught bumble bee gynes, about 30–35 nests could be expected using either method. Thus, it may be beneficial for future studies to test the effect of using honey bees with a larger number of replicates to determine if honey bee can make a significant difference.

The decision to use one technique over the other will certainly depend on several logistical factors. With a cofounded system, the amount of space required for nest establishment is half that of single gyne nests and it does not require access to newly emerged honey bee workers. However, whereas gynes are limited and sometimes difficult to catch in substantial numbers, the prospect of raising even a few more nests may outweigh the costs associated with using honey bee workers. There also may be some differences among species in nest establishment success using the different methods as well; however, due to the difficulty in obtaining gynes, the sample size of the present study was not large enough to adequately test all of the possible iterations of species by initiation technique.

The inverse correlation between time to nest initiation and reaching maximum colony size may prove a useful metric for determining how long to keep gynes in captivity. The fewer the days to first worker emergence, the larger the eventual colony tended to be. Because each gyne retained in the laboratory, whether accompanied by workers or not, requires pollen and nectar in addition to time devoted to activities such as nest cleaning, it is best to limit the time invested in gynes that are unlikely to produce usable colonies. To reduce inputs into slow-to-grow nests, producers can set time limits that gynes are retained without brood. All of the colonies that produced more than 15 workers had commenced egg laying by the 21st day, regardless of treatment or species. Thus, an alternative treatment of gynes (e.g. combining non-laying gynes into communal boxes, CO^2^ narcosis, or termination) that have not commenced oviposition by that point seems advisable. Regardless of the exact time period allotted to begin nesting, it is prudent to consider a limit when commencing investigations. Further studies of the benefits of each technique and the dynamics of nest initiation will be informative for developing commercially viable species.
